# Terahertz-readable laser engraved marks as a novel solution for product traceability

**DOI:** 10.1038/s41598-023-39586-5

**Published:** 2023-08-01

**Authors:** Pouria Hoveida, Adrian Phoulady, Hongbin Choi, Nicholas May, Sina Shahbazmohamadi, Pouya Tavousi

**Affiliations:** University of Connecticut, Storrs, CT USA

**Keywords:** Lasers, LEDs and light sources, Techniques and instrumentation, Engineering, Materials science, Mathematics and computing

## Abstract

Counterfeit products pose significant economic, security, and health risks. One approach to mitigate these risks involves establishing product provenance by tracing them back to their manufacturing origins. However, current identification methods, such as barcodes and RFIDs, have limitations that make them vulnerable to counterfeiting. Similarly, nonvolatile memories, physically unclonable functions, and emerging techniques like Diamond Unclonable Security Tag and DNA fingerprinting also have their own limitations and challenges. For a traceability solution to gain widespread adoption, it must meet certain criteria, including being inexpensive, unique, immutable, easily readable, standardized, and unclonable. In this paper, we propose a solution that utilizes ultrashort pulsed lasers to create unique, unclonable, and immutable physical tags. These tags can then be read nondestructively using far-field Terahertz (THz) spectroscopy. The primary objective of this paper is to investigate the feasibility of our proposed approach. We aim to assess the ability to distinguish laser marks with varying depths, evaluate the sensitivity of THz reading to laser engraving parameters, examine the capacity to capture high-information-density marks, and explore the ability to capture subsurface tags. By addressing these aspects, our method holds the potential to serve as a universal solution for a wide range of traceability applications.

## Introduction

Counterfeit products^1,2^ impose significant economic, security, and health risks on governments, industries, and societies. Counterfeit microelectronics cause billions of dollars in annual losses, while counterfeit pharmaceuticals endanger thousands of lives daily. Avoiding the use of counterfeit products is perhaps the most effective action to mitigate these risks, which requires establishing product provenance by tracing them back to their manufacturing origins. Currently, several techniques are employed to address this issue^3^, including barcodes, passive and active RFIDs, and others. However, despite their partial effectiveness in addressing certain traceability concerns, various challenges persist. One major challenge is that these identification methods themselves are susceptible to counterfeiting^4^. Counterfeiters can easily clone existing identifiers, such as barcodes, to disguise their products as authentic. While more sophisticated methods have been proposed, they often come with high implementation costs, handling difficulties, readout complexities, and usability issues Nonvolatile memories (NVMs), which can be used for chips, are expensive and may not be suitable for smaller chips^5^. The utilization of Nonvolatile memories (NVMs) necessitates the powering up of the device, which becomes impractical when dealing with a large number of parts that require investigation within their packages. Similarly, physical unclonable functions (PUFs), which are also applicable to electronic chips^6^, encounter the same challenge of requiring device power-up and have additional limitations regarding the storage of information. In fact, while PUFs enable the creation of a unique signature, they do not permit the selective embedding of data onto the device. Newly emerging methods also come with their own set of challenges. For instance, the diamond unclonable security tag (DUST) is a trending method that provides a tamper-proof identity for physical items by utilizing quantum-engineered diamond nanocrystals embedded in high-performance polymers^7^. However, this solution is expensive and poses difficulties in implementation. Additionally, it is not envisioned as a practical solution for application at the chip level, thereby resulting in a disrupted traceability of the chain of custody. Similarly, DNA fingerprinting solutions, such as the one developed by Haelixa, encounter similar challenges when applied to microelectronics^8^. In general, an effective traceability solution has to meet the following criteria so that it can be widely adopted by industries and governments as a means to overcome the existing counterfeit issues: (1) Embedment of identifiers in products should be inexpensive; (2) Identifiers must be unique; (3) Identifiers must be immutable in the sense that any attempt to mutate them must be identifiable and causing destruction of the identifier; (4) Identifiers must be easily readable, preferably in a passive fashion (i.e., no need for power up) to be suitable for field and high-volume applications; (5) Identifiers must be standardized so that they can be adopted widely, which is key for their effectiveness; and (6) Identifiers must be unclonable. We introduce a novel approach that can potentially address all of the criteria listed above. The proposed technique utilizes ultrashort pulsed laser for creating unique, unclonable, immutable physical tags, in a rapid and inexpensive fashion. It further utilizes far-field Terahertz (THz)^9^ spectroscopy for reading surface and subsurface tags in a non-destructive fashion. The focus of this paper is investigating different aspects of the feasibility of the described method, towards developing a universal solution for a wide range of traceability applications. The feasibility of time-of-arrival THz imaging for distinguishing laser marks with different depths and the resolution of such readings is investigated. Furthermore, the sensitivity of the THz reading to the laser engraving parameters that have been used for creating the mark is studied. The ability of the proposed method for creating high-information-density marks (i.e., large amounts of data per unit area) is assessed by investigating the feasibility of capturing a surface profile, consisting of regions with different height values. Finally, the ability of the THz reading method for capturing subsurface tags is explored.

## Materials and methods

### Method overview

**Figure 1:**
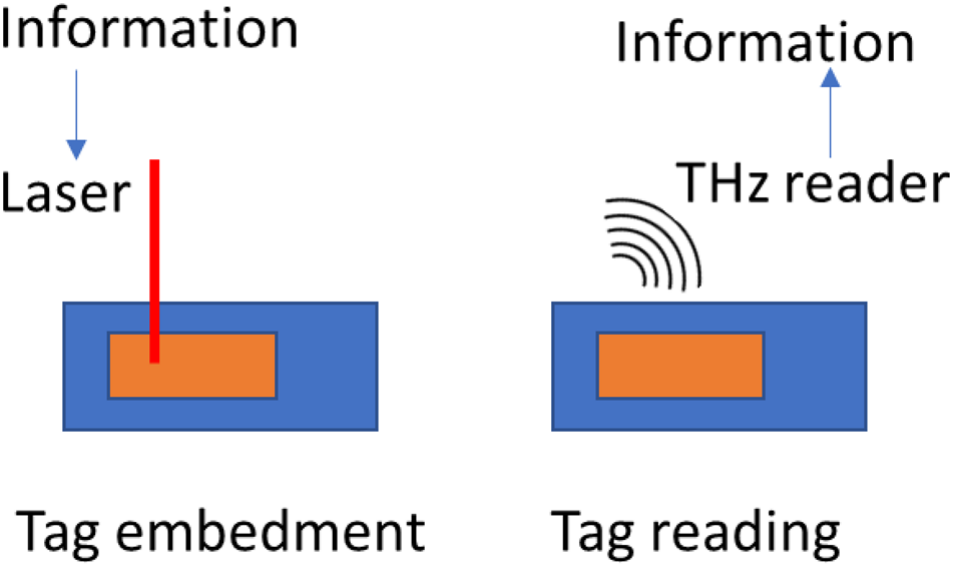
Overview of the proposed traceability method: THz readable laser-engraved identifiers.

The proposed method consists of using ultrashort laser for creating high-information density identifier tags and Terahertz spectroscopy for reading them. This is schematically demonstrated in Fig. 1.

Using different combinations of lasering parameters, tags with different structural properties are obtained. These tags are multimodality readable, where the tag structure can be retrieved at different resolutions using confocal and X-ray microscopy, in addition to THz. Further, a key feature of these tags is that they are unclonable, which is achieved due to the complexity associated with creation of the tags. Each tag is uniquely created using a specific combination of a set of lasering parameters, which is impractical to decode by looking at the structure of the tag, due to the complex and unknown relationship between lasering parameters and the exact resulting tag structure as well as due to the fact that there are too many laser combination possibilities that need to be tested, in a brute force approach, for decoding the recipe for the creation of these tags. It is important to note that the resolution of different reading methods is different in discerning the tag structure variations. Therefore, different reading methods have different sensitivities to the variation of lasering parameters that are used for tag formation. This means that, depending on the size order of the structural variations, higher-resolution reading methods (e.g., confocal and X-ray microscopy) may need to be used for identifying the tag counterfeiting attempts, while lower-resolution methods (e.g., far-field THz) may be used for retrieving tag information in the field.

### Laser machining system

**Figure 2:**
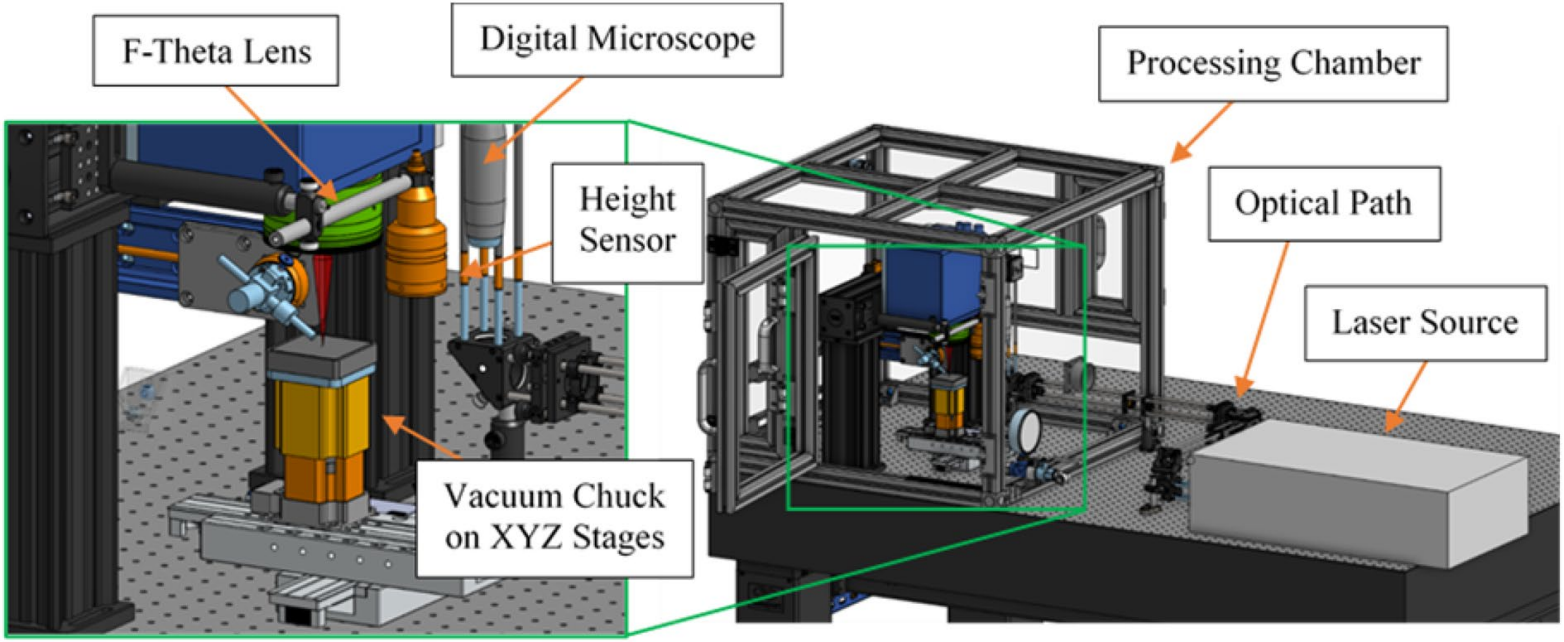
CAD of femtosecond laser machining system.

In this study, the femtosecond machining system employed a Coherent Monaco 1035 nm 40W laser (1035-40-40) with a pulse width of 257 fs. The laser has the capability to generate various pulse repetition rates ranging from single shots to 50 MHz. The emitted beam of the laser has a diameter of 2.7 ± 0.3 mm, which is expanded using a beam expander consisting of a fused silica 75 mm aspherical lens and a fused silica 300 mm convex lens. This expansion results in a beam diameter of approximately 11 mm. The expanded beam is then directed to a telecentric fused silica F-Theta lens (TSL-1064-10-56Q-D20) with an effective focal length of 70 mm. This lens configuration provides a theoretical spot size of around 8.5 μm. Figure 2 provides a computer-aided design (CAD) illustration of the laser setup used in the study.

The femtosecond machining system workflow begins by targeting the region of interest (ROI) with a Dinolite digital microscope (AM73915MZT) and moving the sample accurately on an XY plane using Zaber LDA series stages (LDA150A-AE53T10A). Next, the Keyence confocal height sensor (CL-P070) measures the sample's height with low micrometer resolution, after which the Zaber VSR series stage (VSR40A-T3A) brings the sample into laser focus. Finally, the sample is transferred under the F-theta lens for laser ablation.

### THz system

**Figure 3:**
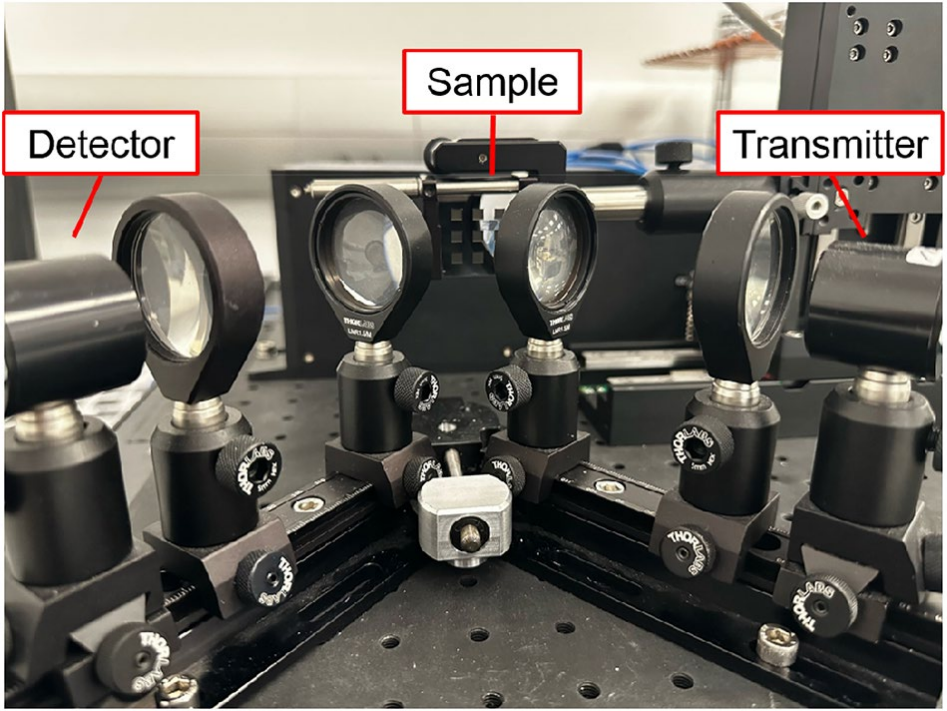
THz reading setup.

THz reflection-mode imaging is used for reading the tags. To obtain the surface profile of the laser-engraved tags, time-of-arrival analysis is conducted. THz beams that are reflected by the lower-height areas on the surface will take longer to arrive at the THz receiver, than those that are reflected by the higher-height areas of surface. This fact is leveraged to create a height map of the tag’s surface structure. Figure 3 shows the used THz setup. Two stages are used to move the sample in x and y directions for scanning.

### Post-imaging analysis

To determine the depth of the sample, the lowest valley of the terahertz time domain spectroscopy signal will be identified for each pixel of the sample image. The location (i.e., time stamp) of this valley indicates the time of arrival of the terahertz signal, which is then used to build a height map of the sample. Since the obtained values correspond to the distance of the wave path from the source to the sample and back to the detector, any tilting of the sample would result in a skewed height map. To address this issue, the height map is levelled to ensure accuracy in the depth measurement of the sample.

## Results and discussion

**Figure 4:**
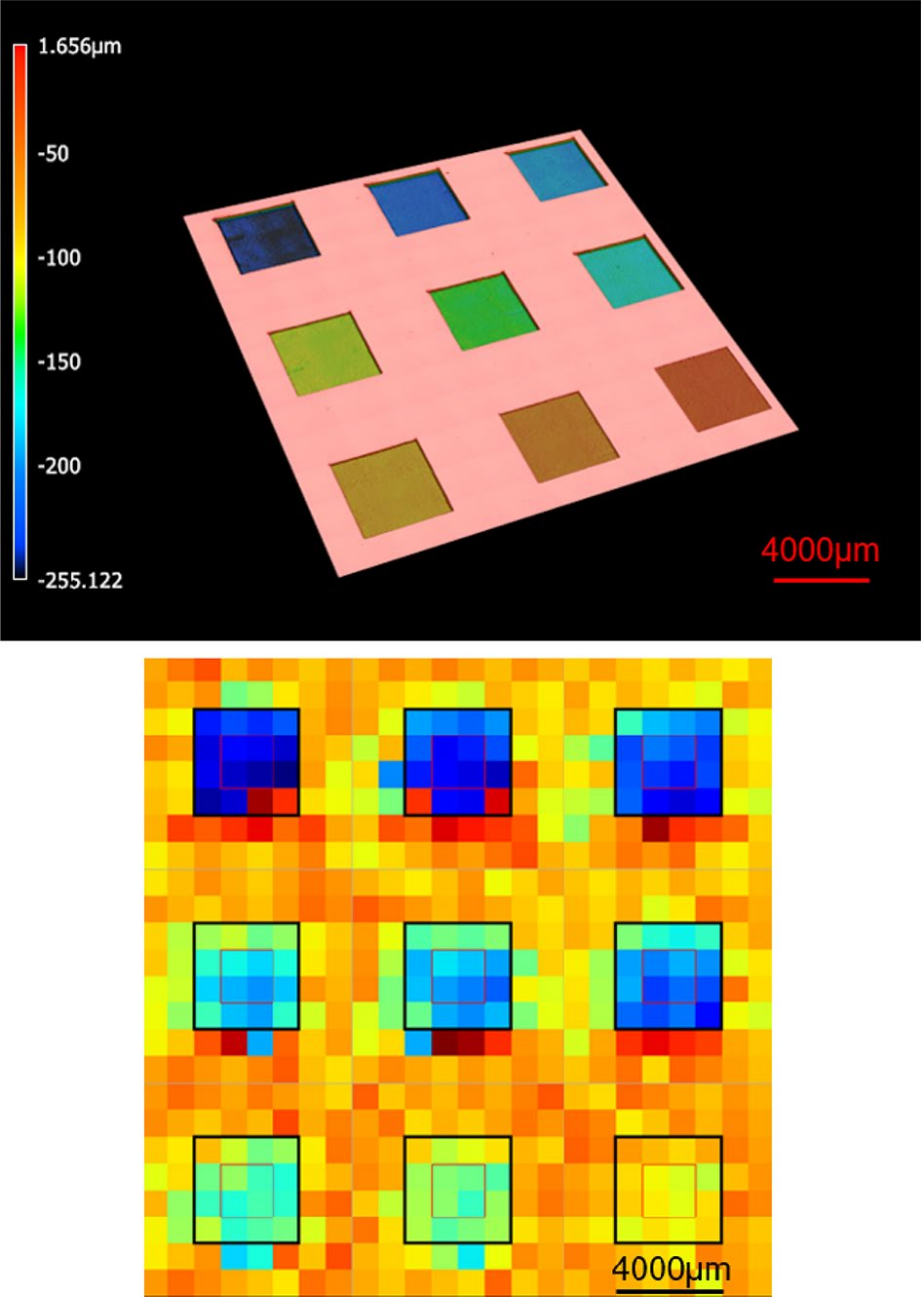
Top: Color-coded confocal height map of one example wafer sample; Bottom: Color-coded time of arrival. The color code for the bottom image has a different scale from the top image.

**Figure 5:**
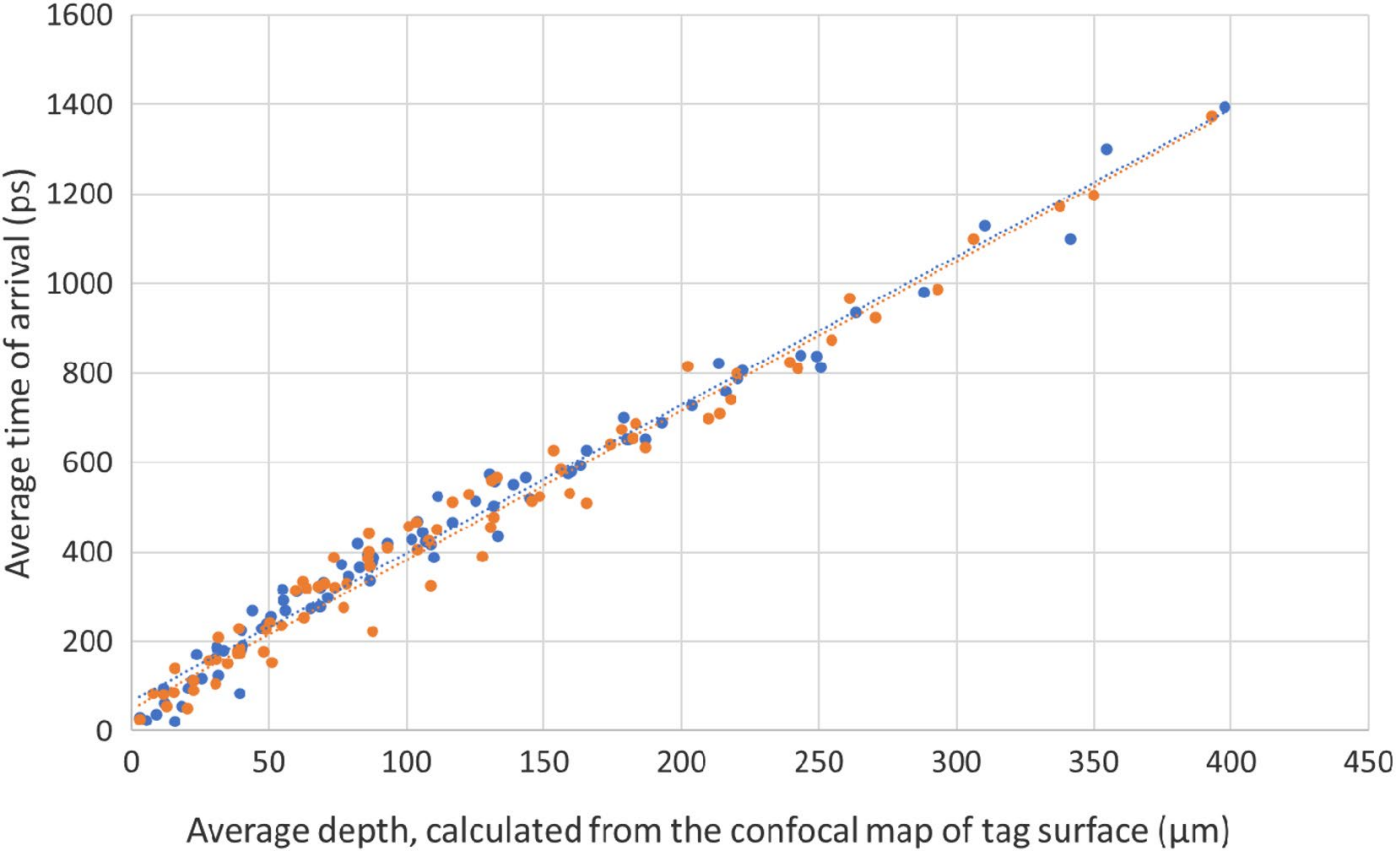
Average time of arrival calculated from the processed THz signal of the imaged trenches, against their average depth as calculated from the confocal height map for the 81 trenches. Blue and orange colors indicate the two repeats. Dotted lines are trendlines for the two repeats.

Several experiments were conducted to explore the feasibility of the proposed approach, namely reading laser-engraved tags using THz. Given that THz time-of-arrival was chosen as the method for reading the tags, the initial experiment focused on assessing the ability to distinguish between surface structures with varying average depths. A total 162 trenches were created on 18 silicon wafer samples (each sample containing 9 trenches) using the laser machining system. As for laser parameters for these engraving trenches, 81 different combinations were used, resulting in 2 repeats for each combination for a repeatability investigation. The size of each wafer sample was 3 cm × 3 cm and the size of each trench was 4 mm × 4 mm. The used laser parameters were as follows: (1) Repetition rates of 250 kHz, 500 kHz, and 1 MHz; (2) Number of lasering cycles between 20 and 180; and (3) Laser power between 1W and 6.97W. The average time of arrival for the trenches was measured using the THz-time domain spectroscopy (TDS) system in reflection mode and their average heights was assessed with laser confocal microscope for a correlative study. Figure 4 shows one wafer sample with the engraved trenches, for which the height map has been obtained using both confocal and THz-TDS systems. Figure 5 plots the offset, average time of arrival calculated from the processed THz signal of the imaged trenches, against their average depth as measured from the confocal height map.

As can be seen in the chart, there is a linear correlation between the depth of the trench and the time of arrival in the THz reading, which suggest that the depth value, which can be controllably produced by laser, can be used for information storage and retrieval, towards creation of unique identifiers. Further, the extent of deviations from the perfect linear relationship, in Fig. 5, determines the sensitivity of the Terahertz reading to the variations in height.

**Figure 6:**
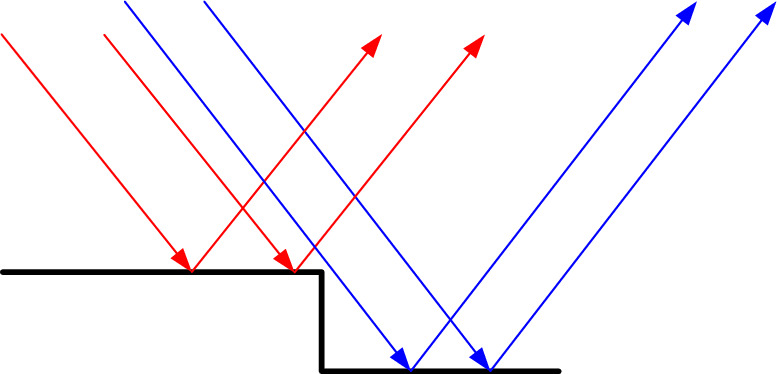
Reflections of different portions of the beam by surfaces at different heights.

The storage of THz-retrievable information in laser-engraved tags needs to be examined in terms of height-reading resolution in both the vertical and lateral directions. According to Fig. 5, THz Time-Domain Spectroscopy (THz-TDS) enables a resolution of 50 μm or better for height measurement in the vertical direction. However, the lateral resolution of Terahertz is relatively low, typically in the order of millimeters. If each pixel is utilized to read only one height value, the resulting lateral resolution would be significantly limited. This is because, out of an information-rich signal collected by focusing the THz beam at one spot, only the time stamp of the lowest valley is extracted to determine the average height. However, if the rather wide THz beam hits a surface in a way that covers both ups and downs, it results in multiple reflections with temporal offsets. This, in turn, produces a THz-TDS signal with multiple major valleys. The time stamps of these multiple identified major valleys correspond to different height values on the surface of interest. Therefore, one pixel can contain more information than just a single height value. To investigate the feasibility of this approach, an experiment was conducted where the edge of a trench was examined using the Terahertz reading system. The edge was positioned at an approximate 45-degree angle relative to the scanning direction, increasing the chances of capturing scenarios where the THz beam covers both sides of the edge (Fig. 6).

**Figure 7:**
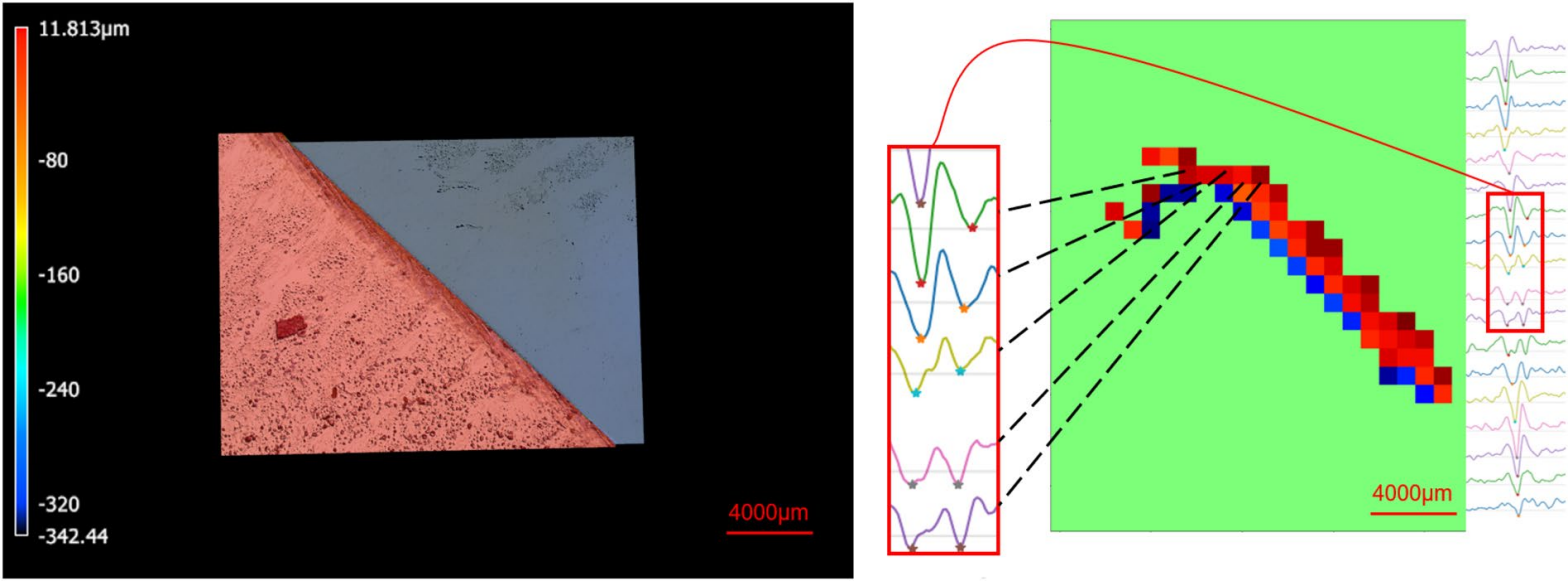
Top: Confocal map of sample with edge; Bottom: Time of arrival (only pixels, whose signal has two valleys).

Figure 7 demonstrates the color-coded confocal map of the sample with an edge as well as the corresponding time of arrival map. It is important to note that, in order to investigate the hypothesis mentioned earlier, only the pixels for which two valleys were detected in the signal are displayed in the time-of-arrival map. All other pixels are colored green. This selective display has resulted in a clear and distinct image of the edge in the time-of-arrival map.

**Figure 8:**
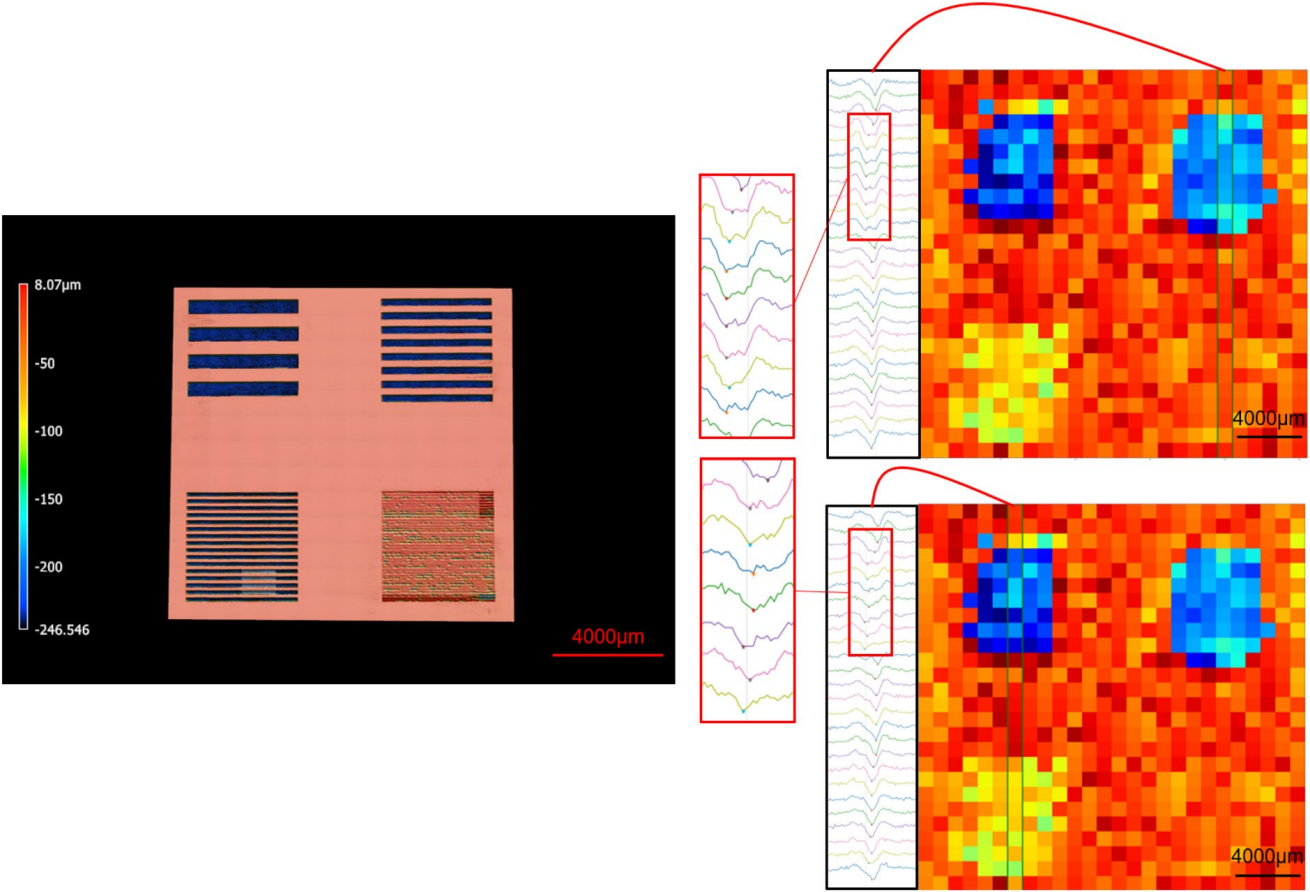
Stripe patterns, measured by confocal microscopy and THz-TDS.

Pushing the limits of the lateral resolution, as described above, was further explored by creating stripe pattern, consisting of consecutive ups and downs, followed by a THz measurement. Four stripe patterns were created. The distance between a low-height region and its adjacent high-height region was 1 mm, 500 μm, 300 μm and 100 μm in these four patterns. Figure 8 shows the confocal map and the THz-TDS signals for these stripe patterns. Presence of two different height values in the sample is clearly observed for in the top two patterns, corresponding to the two major valleys in the THz signal.

**Figure 9:**
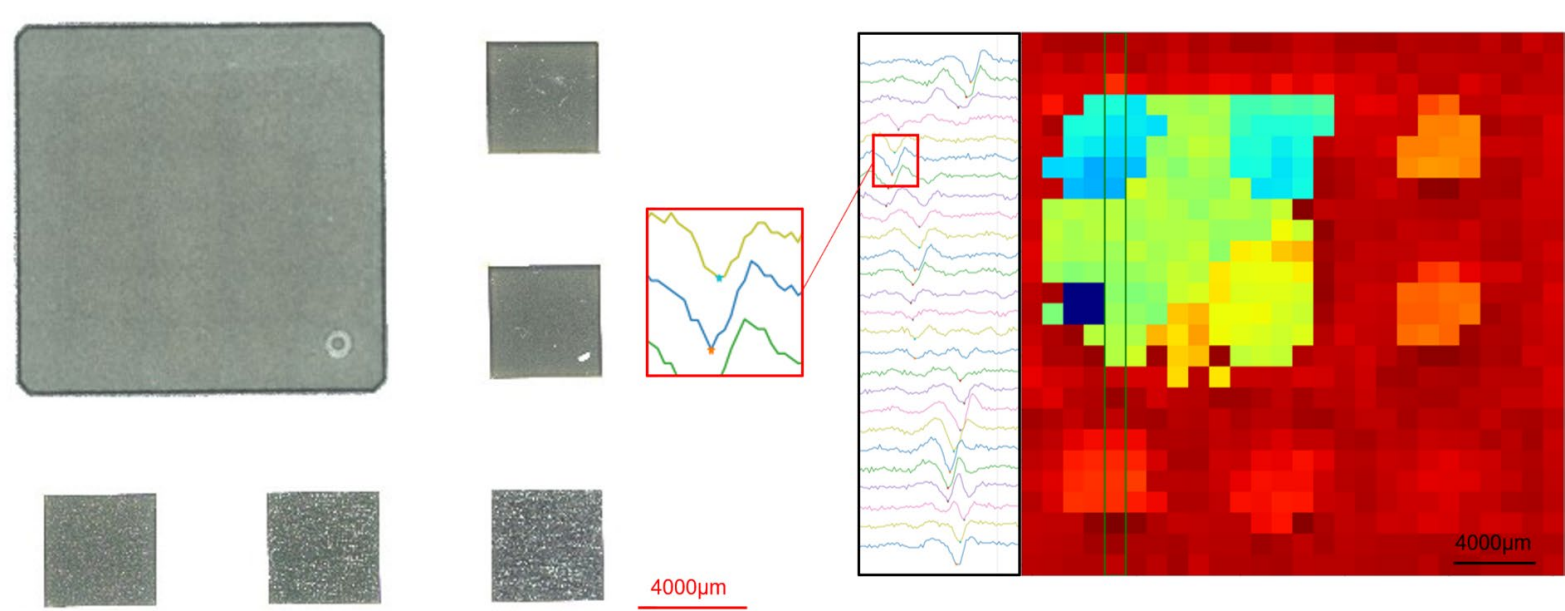
Packaging material covering the four top-left trenches (tags): The buried trench (tag) is still detectable by THz through packaging.

Given that a main target application for the proposed technique is envisioned to be die-level traceability in microelectronics, it must be investigated whether the laser-engraved marks can be read through the packaging material. In order to explore the use of THz for identifier retrieval through packaging, a piece of packaging material was utilized to cover some of the laser-engraved trenches on three silicon wafer samples. One example of such a sample is illustrated in the left image of Fig. 9. The right section of Fig. 9 displays the heat map of arrival times for this sample, along with the time-domain signals for a specific column of pixels, indicated by the vertical rectangle in the heat map. The objective was to investigate whether the correlation between depth and time of arrival still holds in these examples, which would validate the use of THz for identifier retrieval through packaging. Table 1 exhibits the sorted sequence of identifier indices based on depth and time of arrival for the six selected identifiers (the two deepest in each sample). The perfect match observed between the two sorted sequences suggests that THz-TDS is a reliable method for retrieving the height index and, consequently, identifying the tag.

**Table 1:** Selected trenches sorted based on depth and arrival time.

Identifier	Row #	Column #	Sample#	Sorted Indices Based on Average Depth	Sorted Indices Based on Average Time of Arrival	Match?
A	1	1	1	C	C	Yes
B	1	1	2	F	F	Yes
C	1	1	3	B	B	Yes
D	1	2	1	E	E	Yes
E	1	2	2	A	A	Yes
F	1	2	3	D	D	Yes

**Figure 10:**
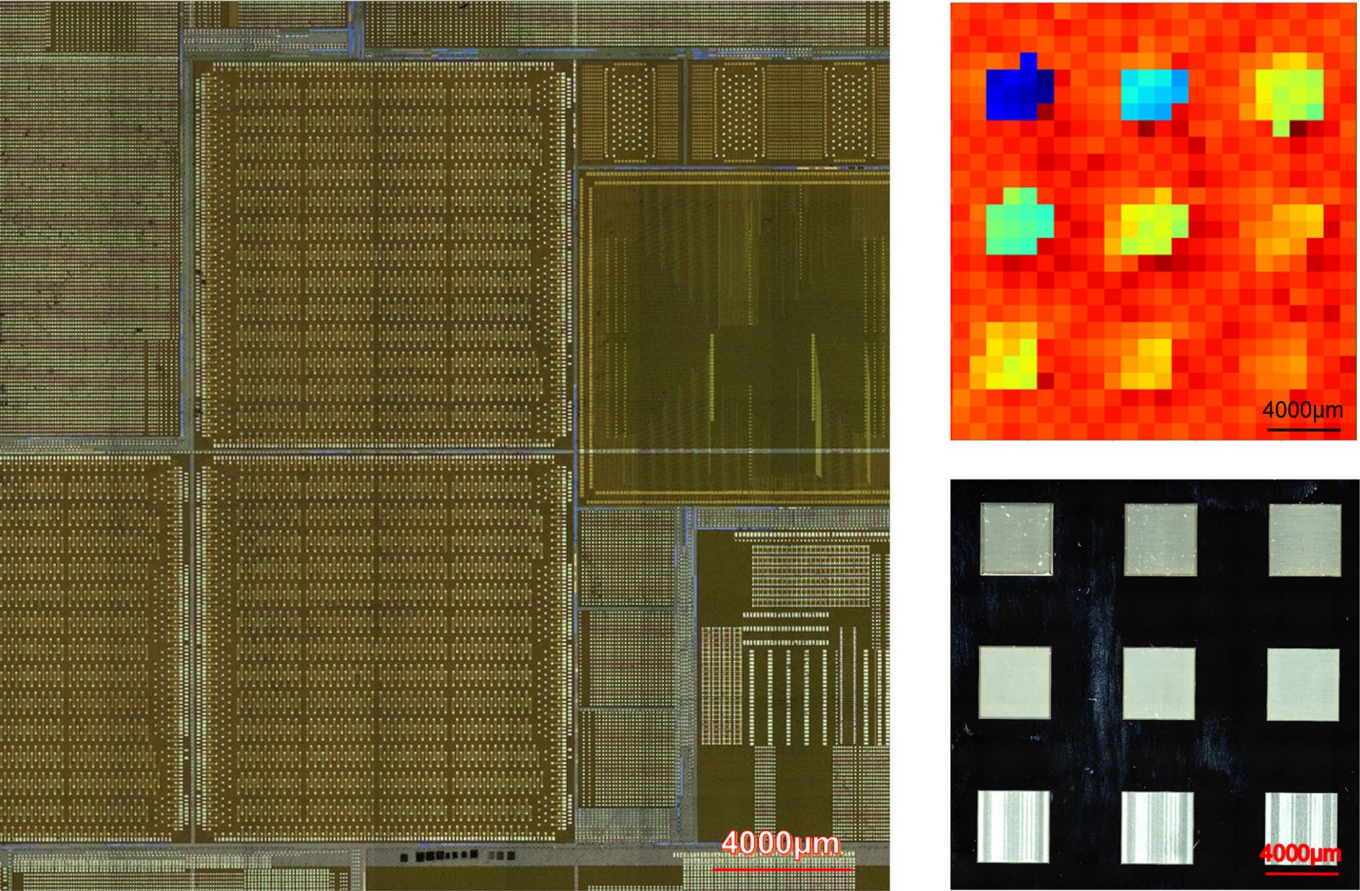
Left: Front side of the die sample; Bottom Right: Back side of the die with engraved square-shaped tags; Top Right: Color-coded time of arrival.

The effectiveness of the proposed method has been verified by embedding physical tags using laser marking on the backside of a die, followed by reading the identifiers using THz. The front side of the die, containing circuitry, is shown in the left section of Fig. 10. Nine square-shaped tags with a range of depth values are created on the back side of the die (Fig. 10 bottom right). The two control parameters for this experiment are number of lasering cycles, which ranges from 40 to 120 and the repletion rate which ranges from 250 kHz to 1 MHz. Table 2 shows the depth values of these 9 tags, characterized using confocal microscopy. Table 2 further has a column showing the sorted sequence of the indices associated with these tags, based on the depth values. Finally, Table 2 has another column showing the sorted sequence of the indices, based on the arrival times, obtained using THz spectroscopy and reflected in the heat map of Fig. 10. There is a perfect match between the two sorted sequences, suggesting that the depth index, which in this example serves as the identifier of these tags, can be retrieved using THz spectroscopy.

**Table 2:** The parameters of physical tags embedded on the back side of a die sample, to serve as unique identifiers.

Tag index	Tag row	Tag column	Number of cycles	Repetition rate	Average depth (μm)	Indices sorted based on average depth	Indices sorted based on average time of arrival (from heat map of height in Fig. 10)	Match?
A	1	1	120	1 MHz	260	A	A	Yes
B	1	2	80	1 MHz	180	B	B	Yes
C	1	3	40	1 MHz	86	D	D	Yes
D	2	1	120	500 kHz	160	E	E	Yes
E	2	2	80	500 kHz	104	C	C	Yes
F	2	3	40	500 kHz	47	G	G	Yes
G	3	1	100	250 kHz	70	H	H	Yes
H	3	2	80	250 kHz	55	F	F	Yes
I	3	3	40	250 kHz	23	I	I	Yes

The presented results serve as an investigation of the capabilities and the limitations of the proposed approach for product traceability. The key novelty of the proposed approach lies in utilizing ultrashort pulsed laser technology to create physical tags in a rapid and cost-effective manner, that can be embedded in products to serve as identifiers, and using far-field THz spectroscopy for reading the information stored in them. This approach aims to address the limitations of existing techniques and provide a potential universal solution for traceability applications in various industries.

The proposed approach is distinguished from existing solutions, including barcodes, RFIDs, non-volatile memories (NVMs), and physical unclonable functions (PUFs), by several advantages. The key advantages deemed for the ultimate form of the proposed solution include: (1) inexpensive embedment of identifiers in products; (2) uniqueness of identifiers; (3) immunity to mutation, with any attempt at altering the identifier causing destruction; (4) easy readability in a passive manner (no need for power-up), suitable for field and high-volume applications; (5) standardization of identifiers for widespread adoption; and (6) unclonability of the identifiers.

Further, it must be emphasized that, although THz spectroscopy has been previously used to characterize surface features in thin films, coatings, and interfaces for understanding their composition, thickness, and optical properties, use of THz for retrieving the identifier of a laser-engraved physical tag is a unique offering of this work.

## Conclusion

Counterfeit products pose significant risks, and one way to mitigate these risks is by establishing product provenance through tracing their manufacturing roots. However, current identification methods have limitations and are susceptible to counterfeiting. In this paper, we have proposed a solution that utilizes ultrashort pulsed laser technology to create physical tags that are unique, unclonable, and immutable. To read these tags in a nondestructive manner, we have employed far field Terahertz (THz) spectroscopy. Our approach holds promise as an inexpensive, standardized, and unclonable solution for a wide range of traceability applications. Throughout the paper, we have demonstrated the capabilities of this method through several examples, showcasing its potential effectiveness and versatility.

## Data Availability

All data generated or analysed during this study are included in this published article.
